# Use Dose Bricks Concept to Implement Nasopharyngeal Carcinoma Treatment Planning

**DOI:** 10.1155/2014/720876

**Published:** 2014-05-21

**Authors:** Jia-Ming Wu, Tsan-Jung Yu, Shyh-An Yeh, Pei-Ju Chao, Chih-Jou Huang, Tsair-Fwu Lee

**Affiliations:** ^1^Department of Radiation Oncology, E-Da Hospital, Kaohsiung 82445, Taiwan; ^2^Department of Medical Imagines and Radiological Science, I-Shou University, Kaohsiung 82445, Taiwan; ^3^Medical Physics and Informatics Laboratory, Department of Electronics Engineering, National Kaohsiung University of Applied Sciences, 415 Chien Kung Road, Kaohsiung 80778, Taiwan; ^4^Department of Urology, E-Da Hospital, Kaohsiung 82445, Taiwan; ^5^Department of Radiation Oncology, Kaohsiung Chang Gung Memorial Hospital and Chang Gung University College of Medicine, Kaohsiung 833, Taiwan

## Abstract

*Purpose*. A “dose bricks” concept has been used to implement nasopharyngeal carcinoma treatment plan; this method specializes particularly in the case with bell shape nasopharyngeal carcinoma case. *Materials and Methods*. Five noncoplanar fields were used to accomplish the dose bricks technique treatment plan. These five fields include (a) right superior anterior oblique (RSAO), (b) left superior anterior oblique (LSAO), (c) right anterior oblique (RAO), (d) left anterior oblique (LAO), and (e) superior inferior vertex (SIV). Nondivergence collimator central axis planes were used to create different abutting field edge while normal organs were blocked by multileaf collimators in this technique. *Results*. The resulting 92% isodose curves encompassed the CTV, while maximum dose was about 115%. Approximately 50% volume of parotid glands obtained 10–15% of total dose and 50% volume of brain obtained less than 20% of total dose. Spinal cord receives only 5% from the scatter dose. *Conclusions*. Compared with IMRT, the expenditure of planning time and costing, “dose bricks” may after all be accepted as an optional implementation in nasopharyngeal carcinoma conformal treatment plan; furthermore, this method also fits the need of other nonhead and neck lesions if organ sparing and noncoplanar technique can be executed.

## 1. Introduction 

External radiation therapy (RT) of nasopharyngeal carcinoma (NPC) typically involves bilateral parotid gland and leads to xerostomia of varying degree [1–13]. High radiation dose to the salivary glands causes a marked reduction in oral saliva output, the degree of which depends on how much of the salivary gland volume irradiated and the radiation dose to these organs. Around the dose of about 45 Gy the hyposalivation may be reversible [14–16], whereas higher sides generally produce irreversible destruction of the salivary glands with permanent dryness [17–19]. The purpose of this study is to minimize xerostomia in patients and focus on the dose to target volume after receiving bilateral head and neck irradiation by using dose bricks concept technique planning to spare a significant volume of bilateral parotid gland from radiation.

There are at least two dose delivery treatment planning techniques to approach the goal of reducing normal organs dose up to now [20–23]. One of the approaches has been proposed frequently that employs intensity-modulated radiation therapy (IMRT) [24–30]. The IMRT is a highly mathematical theory and computerized integration dose delivery technique [31]. This technique, no matter, uses step and shoot or dynamic MLC for dose delivery [32–35]. The critical organs are passed through and obtain dose when the target was irradiated. The undesirable critical organs doses are a flaw that spoils the perfection of this technique. This phenomenon caused a pretty high critical organs dose especially of bilateral parotid glands in bell shape nasopharyngeal carcinoma cases. The second of the treatment technique is three-dimensional conformal therapy [36–38]. Three-dimensional conformal therapy usually could not achieve the goal of high dose distribution to the target and minimum dose to the normal organs in treating retropharynx and parapharynx involved especially bell shape nasopharyngeal carcinoma cases; however, the dose bricks concept treatment planning technique described here can reduce the dose to healthy tissues and sensitive structures such as parotid glands to much lower dose when compared to that of conformal therapy and IMRT result.

Dose bricks concept radiation therapy is a treatment planning technique designed to shape the spatial distribution of the high radiation dose to conform to the target volume, thereby reducing the dose delivered to the normal organs.

This concept includes four major parts: (1) beam shape is fabricated to minimize critical organs damage and focus the maximum dose on target. (2) Planning system must have the function to prescribe dose individually to each field (3) and be normalized to only one normalization point while summering total dose contributed by every individual field. (4) Using nondivergent central axial plane creates dose bricks. The comparison was also made to check dose distribution in between dose bricks method in conformal radiation therapy and IMRT technique.

## 2. Materials and Methods

The study was carried out using Pinnacle (version 8.9) treatment planning computer system. The patient was first simulated in supine position with a Polyflex II immobilizer. CT scans were then taken for images acquisition. The CT data sets were acquired via network for ADAC treatment planning system. Metal wires were put on patient as a land mark during the CT scan. After the treatment plan, the patient underwent a second simulation with treatment plan isocenter setting onto the patient. This procedure was well known as recheck. Elekta precise linear accelerator was employed as dose delivery facilities and 6 MV photon beam was benefits of beam sharpness in head and neck cancer. Kodak O-mat films were used to check the mechanical isocentric characteristics when couch and gantry rotated statically during the dose delivery. The dose delivery accuracy was verified by TLDs. PTW 23333 Farmer type 0.6 cc chamber, keithley 35617 electrometer, PTW 30329 parallel plate chamber, and polystyrene solid phantom were used for output measurement (cGy/mu). Rando phantom was used for mechanical isocenter check and used as dose delivery verification check devices.

Dose bricks idea was an inspiration drawn from the toy blocks; if the radiation beam can be fabricated like a brick then dose can be piled up to the lesion with minimum damage to the surrounding critical organs. Treatment planning isocenter was located along patient's longitudinal axis towards superior direction at about 1.5 cm away from the last superior lesion CT slice and was set at tumor bilateral geometry center. There are at least five noncoplanar fields to create dose bricks technique treatment plan. Each field was one fourth quadrant of full field and dose brick was created by one of the central axial planes as a nondivergent abutting field. Fields numbers, shape, and geometry positions should be modified depending on the lesion size and location. The five fields are (a) right superior anterior oblique (RSAO), (b) left superior anterior oblique (LSAO), (c) left anterior oblique (LAO), (d) right anterior oblique (RAO), (e) superior inferior vertex (SIV).

Treatment plan was proceeded to design the five fields dose bricks technique once the isocenter has been defined. All five fields are described in detail as follows.

### 2.1. Right Superior Anterior Oblique (RSAO)

**Figure 1:**
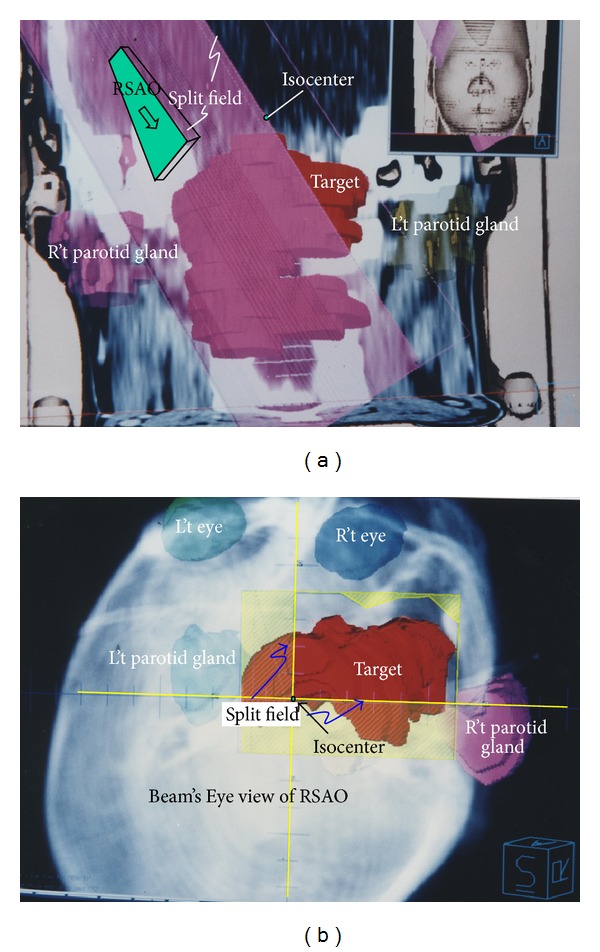
(L't) Picture shows the split field of right superior anterior oblique (RSAO) field in coronal plane. The red color wash area in the center of this picture represents the target. Right and left side parotid glands were represented in pink and brown color, respectively. Both right and left side parotid glands were spared with block. In this field, couch was rotated to 120°, gantry was rotated to 260°, and collimator was rotated at degree of 180°. 60-degree wedge with “thick head” toward gantry side was added for dose uniform modification. (R't) Picture shows the Beam's Eye View (BEV) in Digital Reconstruction Radiograph (DRR) of RSAO field. RSAO beam shape (yellow color) covers most of the lesion. There is two-field edge with no divergence, horizontal and vertical field edge along the central axis plane. Both right and left side parotid glands were spared by asymmetry closing jaw.

The field direction was defined basically on patient's outward appearance. There are six beam's direction definitions: right, left, anterior, posterior, superior, and posterior. For example, right means the beam comes from patient's right side and superior means the beam comes from head, and so forth. The couch, gantry, and collimator angle definition in IEC scale is as follows: couch longitudinal parallel to beam radial direction is 180°, counter clockwise to 90°, and clockwise to 270°. Collimator tray holder open toward couch was defined to 180°; collimator tray holder open toward 90° couch and 270° couch represent 90° and 270°, respectively. The gantry angle with beam axis toward ground means 180° while gantry rotates clockwise to 90° and rotates counter clockwise to 270°. The first beam was set from patient's right superior to anterior obliquely and was named RSAO. Right side parotid gland was spared with MLC while left side parotid gland was spared with split field (Figure 1(a)). In this field the couch was rotated to 120°, the gantry angle was 260°, and collimator was set at an angle of 180°. A sixty-degree wedge with “thick end” toward gantry side was added for dose uniform modification. Beam's Eye View in Digital Reconstruction Radiograph (DRR) showed how the critical organs were spared (Figure 1(b)).

### 2.2. Left Superior Anterior Oblique (LSAO)

**Figure 2:**
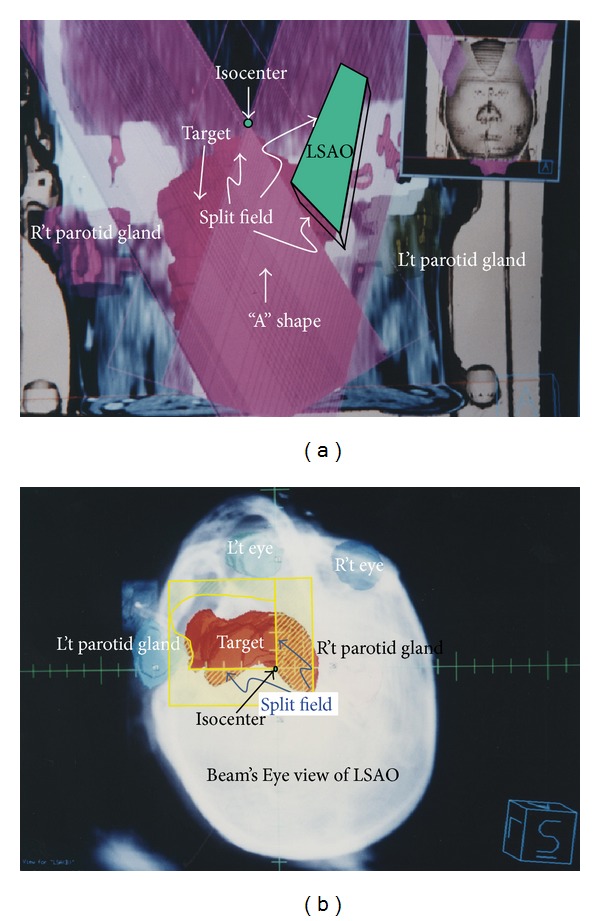
(L't) Left superior anterior oblique (LSAO) field was added to create an “A” shape dose overlap region (overlapping of RSAO and LSAO). This picture shows the split field of LSAO in coronal plane. Right and left side parotid glands were represented in pink and brown colors, respectively. In this field, couch was rotated to 250°, gantry was rotated to 98°, and collimator was rotated at degree of 172°. Sixty-degree wedge with “thick head” toward gantry side was added for dose uniform modification. (R't) This picture shows the Beam's Eye View (BEV) in Digital Reconstruction Radiograph (DRR) of LSAO field. Two central axis nondivergence planes (denoted as “split field” on the left hand side) are ready to be abutted by RAO (vertical) and SIV (horizontal) fields.

After RSAO field has been defined, left superior anterior oblique (LSAO) was added to meet an overlap shape “A” as in Figure 2(a)). Right parotid gland was spared with split field while the left parotid gland was spared with a MLC in DRR (Figure 2(b)); the couch was rotated to 250°, gantry was rotated to 98°, and collimator was rotated to 172°. Sixty-degree wedge with “thick end” toward gantry was added to achieve dose uniformity.

### 2.3. Left Anterior Oblique (LAO)

**Figure 3:**
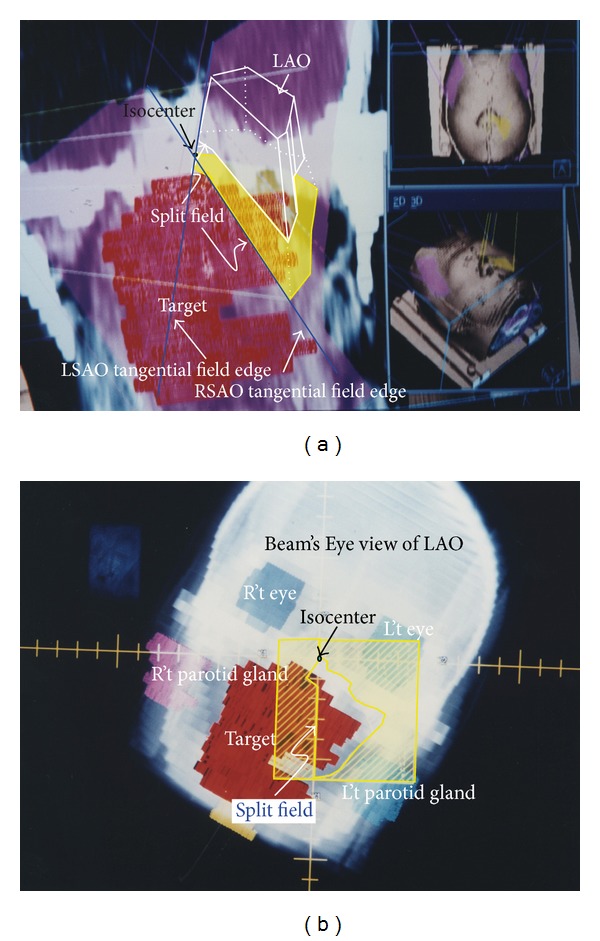
(L't) Left anterior oblique (LAO, yellow color area) field was used to compensate the target dose deficient at left side where it is shown outside the “A” shape at upper right on this figure. The yellow color wash region represents the field aperture of LAO on coronal plane and on patient surface. The central axis plane abutted the interface of “A” shape and LAO, which was created by rotating the collimator to 153°, gantry angle to 180°, and couch rotation to 180°. Forty-five degree wedge with “big head” toward R't side was added for dose uniform modification. (R't) This picture shows the Beam's Eye View (BEV) in Digital Reconstruction Radiograph (DRR) of LAO field. This field was created essentially for compensating the target dose deficiency right side to “A” shape. The interface close to “A” (denoted as “split” abutted onto A shape edge on this figure) was opened by jaw to form nondivergence field.

The fields RSAO and LSAO created a dose overlapping region with an “A” shape; however, the dose deficiency region in both right and left sides should be compensated by the beam coming from anterior direction. The part of lesion excluded by RSAO irradiation beam was irradiated by left anterior oblique (LAO) in Figure 3(a) to compensate the dose deficiency in this field. Left side parotid gland was excluded in this field, and the interface of “A” field and LAO was abutted by the central axis plane by rotating the collimator to 153°, gantry to 180°, and couch at the degree of 180°. A forty-five degree wedge with “thick end” toward patient right side was added for dose uniform modification. Beam's Eye View in DRR showed how the critical organs were spared (Figure 3(b)).

### 2.4. Right Anterior Oblique (RAO)

**Figure 4:**
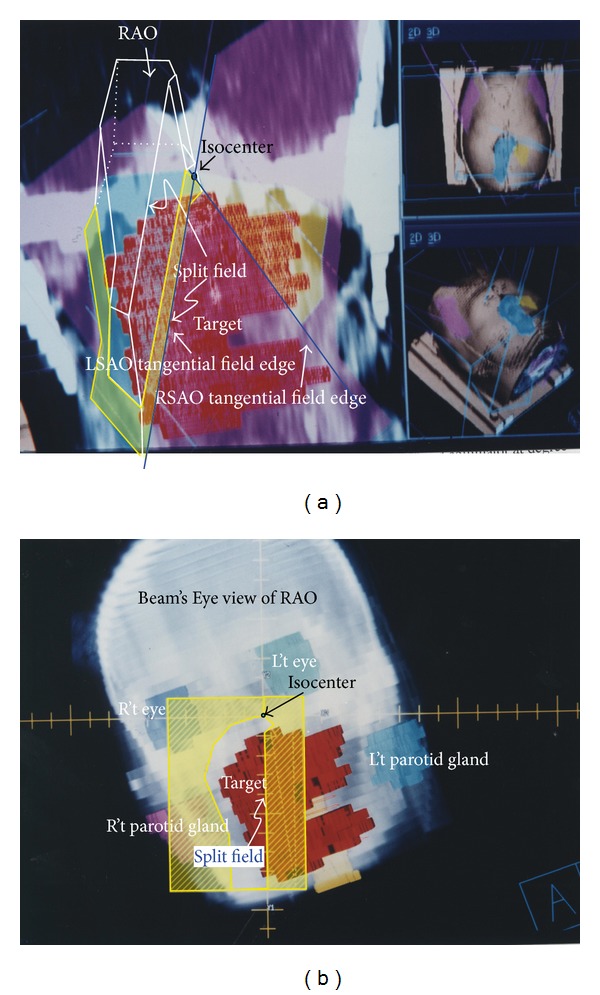
(L't) Treatment couch was set to 180°, gantry to 172°, and collimator rotation at degree of 199° to create RAO field (yellow meshed area on coronal plane and blue sky color area on patient surface) to compensate the nonoverlap dose deficiency lesion region on left side area (left side on picture). The gantry 172° means the beam direction comes from patient's left side to right side obliquely when the patient was set in supine and with head toward gantry position. Basically, this portal is a little left anterior oblique incidence to patient. (R't) The DRR of RAO field. The interface close to “A” (denoted as “split” abutted onto A shape edge on this photo) was opened by jaw to form nondivergence field.

The field LAO was created after RSAO and LSAO to form a uniform dose overlapping region and thus right side still has an under dose region in Figure 4(b). Right anterior oblique (RAO) was added to compensate the nonoverlap region (Figure 4(b)). In this field, couch was set at 180°, gantry at 172°, and collimator at degree of 199°. All the fields' directions were defined basically on patient's outward appearance except RAO. According to IEC scale, gantry 172° means the beam direction comes from patient's left side to right side obliquely with the position of supine and patient head toward gantry. Basically, this portal should be named left anterior oblique and will be probably confused with former LAO. So simply give it the name RAO. Forty-five degree wedge with “thick end” toward patient left side was added to achieve dose uniformity. Beam's Eye View in DRR also showed how critical organs were spared in Figure 4(a).

### 2.5. Superior Inferior Vertex (SIV)

**Figure 5:**
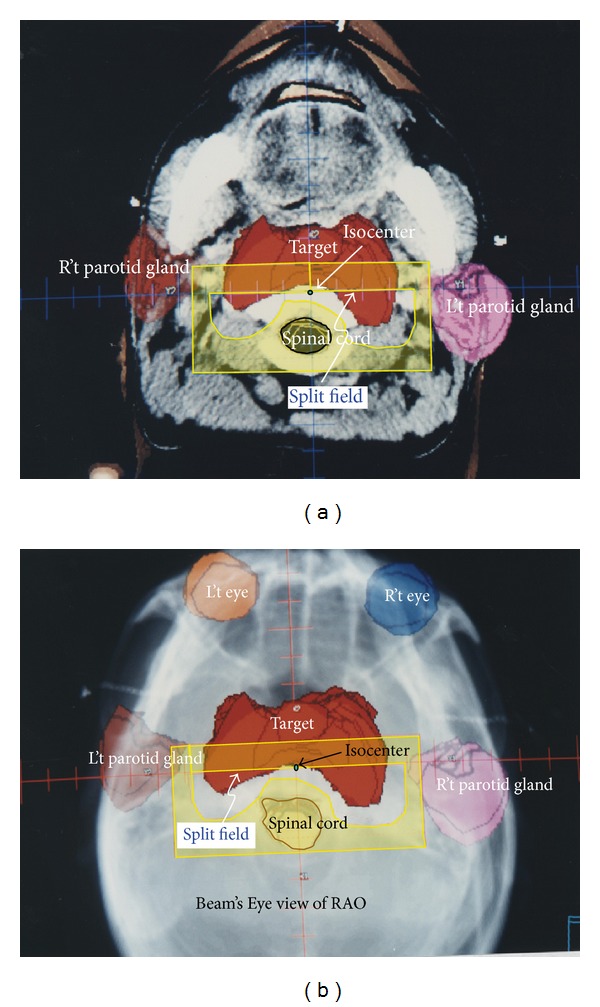
(L't) The lower portion of bell shape was compensated by superior inferior vertex (SIV) field due to RSAO, LSAO, LAO, and RAO contributing insufficient dose to this region. (R't) The BEV in DRR of SIV field. The interface abutted onto RSAO and LSAO was created by jaw opening.

RSAO, LSAO, LAO, and RAO created good geometry coverage on most lesions except the bell shape lower portion in Figure 5(b). The superior inferior vertex (SIV) was added to compensate the lower portion of the bell shape. In this field couch was rotated to 90°, gantry to 261°, and collimator at the angle of 175°. Beam's Eye View in DRR showed how we delineated the block shape and spared the critical organs in Figure 5(a). Because the dose of lower portion of bell shape was partially contributed by LAO and RAO, the prescribed dose of SIV was only 25 cGy.

**Figure 6:**
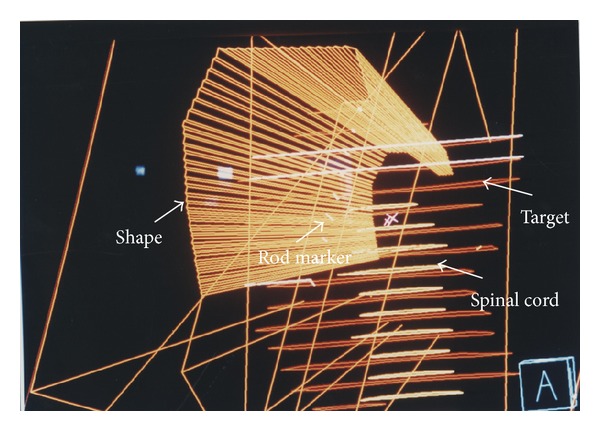
A set of rod markers were used to check the beam direction. These rod markers (white color in picture) were parallel to the central axis plane. If gantry angle was in the wrong direction the rod becomes a bar instead of a point.

**Figure 7:**
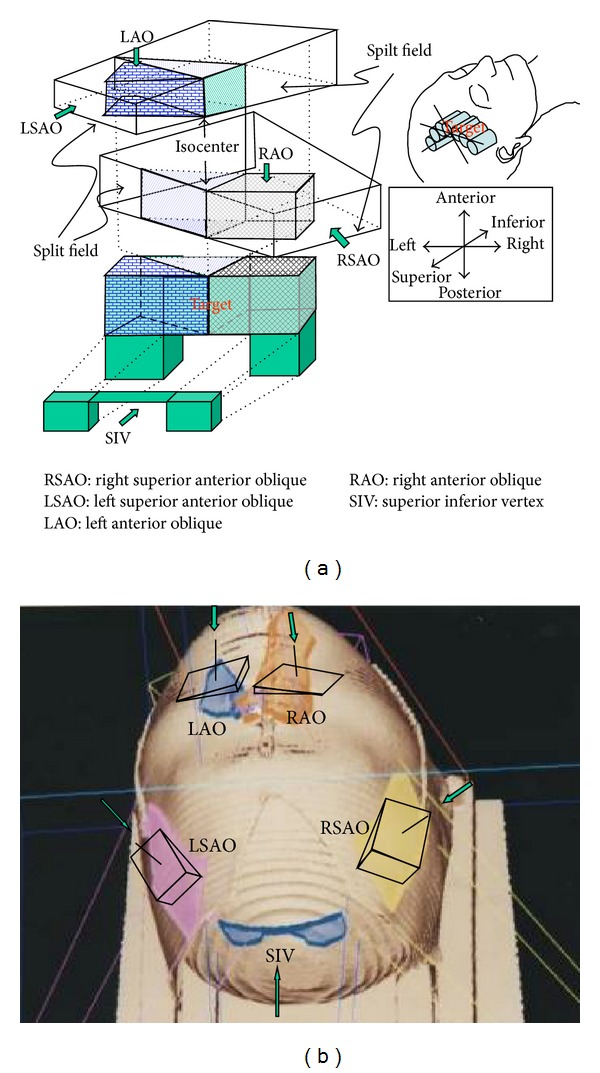
The upper photo illustrates how these 5 beams form dose bricks onto the patient and lower picture shows these five fields outward appearance with wedge orientation on patient surface.

The dose calculation grid geometry was 0.4 cm × 0.4 cm × 0.4 cm pixel by pixel. Field abutting was checked by marking a series of rod markers in Figure 6. These rod markers were parallel to the central axis plane. If gantry angle was in the wrong direction the rod marker becomes a bar instead of a point. All of these fields were abutted by the jaw end, so there are no divergences overlapping in every field. Illustration of cartoon in upper part of Figure 7 shows how this field piles up the dose bricks and these five fields outward appearance on patient surface were shown in the lower part of Figure 7. The parameters of these five fields were listed in Table 1. The prescribed dose in this table means the dose prescription to each field and is normalized to reference point individually.

### 2.6. Output Calibration

The external radiation field output was calibrated by Farmer 0.6 cc ion chamber, PTW parallel chamber according to TG-21 protocol [31]. For small field such as SIV, the output was checked by TLDs and scintillation detector. TLDs or scintillation detector was embedded 5 cm deep in solid phantom for small field and compared the dose to that of external beam in the field size of 10 cm by 10 cm to know the exact output of small field used in DB technique.

### 2.7. Penumbra of Abutting Field

Two half beam fields were abutted onto the central axis plane field in air in three conditions: (1) with 1 mm overlap (2), without any separation, and (3) with 1 mm separation to check the penumbra performance in this region.

### 2.8. Mechanical Rotation Isocenter

**Figure 8:**
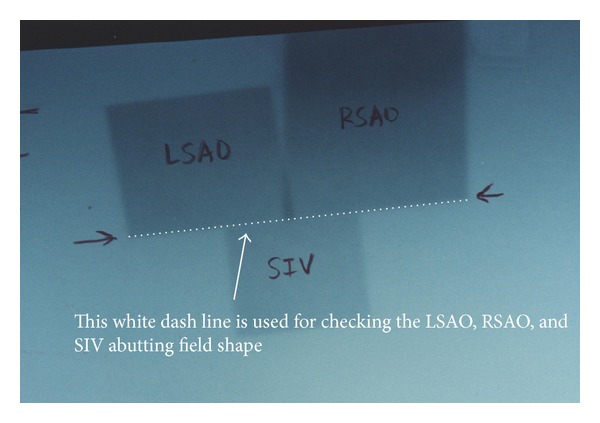
Mechanical isocenter checks of SIV, RSAO, and LSAO field in dose brick technique. Film was put at the isocenter, perpendicular to treatment table top and exposure by RSAO, LSAO, and SIV. The opposite arrow sign can be used to check the performance of machine mechanical isocenter on the junction (white dash line) of RSAO, LSAO, and SVI.

**Figure 9:**
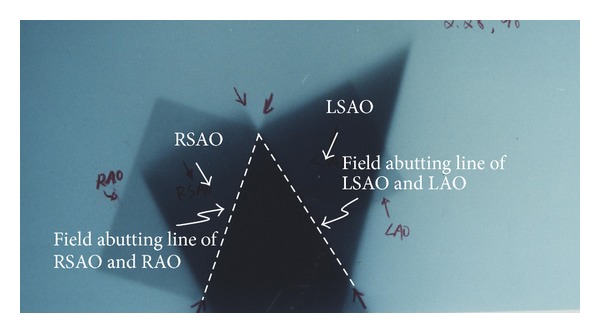
This photo shows the accuracy check of mechanical isocenter and treatment setup. Film was put parallel to treatment table top at the isocenter without any phantom on film and exposure by RSAO, LSAO, LAO, and RAO fields. The two white dash lines can be used to check the abutting junction line of “A” shape to LAO, LSAO, RAO, and RSAO.

The couch, gantry, and collimator were rotated according to this technique planning parameters of all the five fields. So we take verification films with planning condition in air to check the mechanical rotational accuracy in the treatment room. The film was put parallel to the transverse direction with plane vertically to the ground and exposed by the RSAO, LSAO, and SIV fields to check the field junction (Figure 8). The other film was put with the plane parallel to the couch surface and exposed by RSAO, LSAO, RAO, and LAO to check the field junction (Figure 9).

## 3. Results

**Figure 10:**
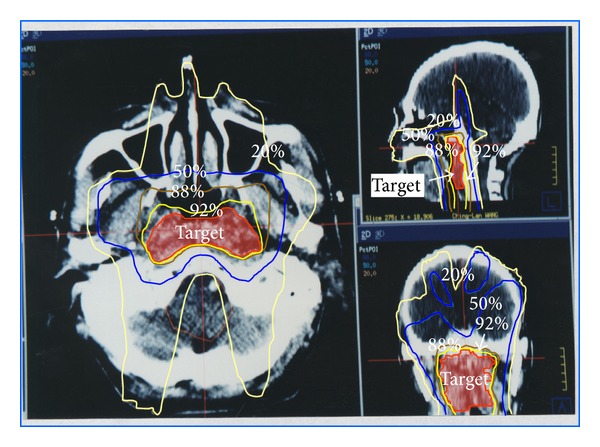
The isodose curve in the transverse, coronal, and sagittal planes for the nasopharyngeal tumor treatment plan in dose bricks technique. The 92% dose curve conforms to the target while 50% dose caused by RSAO, LSAO, and SIV fields cover more brain area than IMRT.

**Figure 11:**
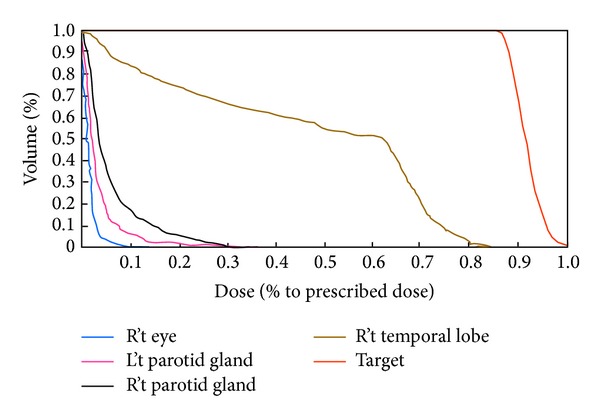
The DVH of DB technique. 50% volume of right parotid gland received less than 10% of prescribed dose.

The isodose curves in the transverse, coronal, and sagittal planes for the nasopharyngeal tumor treatment plan that pass through the centroid as the tumor volume are given in Figure 10. The isodoses are given as a percentage of the isocenter dose of 180 cGy that occurred in the Clinical Target Volume (CTV). The 92% isodose curves can almost cover the CTV while parotid glands are spared in this method. DVH showed that 50% volume parotid glands received less than 10% dose (Figure 11). 50% volume of right temporal lobe received 60% of prescribed dose while half volume of right ear received 50% of prescribed dose. 50% volume of right parotid glands received less than 10% while half volume of right eye received scatter dose which was less than 5% of prescribed dose. A dose of 45 Gy was the presumed threshold for xerostomia.

For small field such as SIV, the output result calibrated by TLDs and scintillation detector for plan calculation MU and measurement of BEV's field were in great agreement.

As for the penumbra of abutting field in air in three conditions, the optic density result reflected the dose overlap performance to every abutting field in air. The results were as follows. (1) the dose converted from optic density derived by H-D curve showed there was about 8–10% dose escalation in 1 mm overlap region. (2) Light and radiation disagreement will result in dose over or under about 2-3% in nongap region. (3) Under dose will come out about 10–12% in the 1 mm separation region.

The optic density in abutting line of fields RSAO, LSAO, and SIV (arrow sign point oppositely, Figure 8) indicates the coincidence of two nondivergent central axis junction planes. The optic density in abutting line of field LAO next to shape “A” as well as the field RAO next to shape “A” also indicates the coincidence of two sets of nondivergent central axis junction plane (Figure 9). The mechanical isocenter in good condition can result in accuracy of dose delivery in treatment planning.

## 4. Discussion

Intensity-modulated radiation therapy (IMRT) is an advanced mode of high-precision radiotherapy that uses computer-controlled linear accelerators to deliver precise radiation doses to a malignant tumor or specific areas within the tumor. IMRT allows for the radiation dose to conform more precisely to the three-dimensional shape of the tumor by modulating or controlling the intensity of the radiation beam by computerized treatment planning system calculation and dose delivery through Multileaf Collimator. The method described in this paper is to expound another method for treating H & N tumor (e.g., NPC) other than standard IMRT technique. This method provides the other choice and simple way to archive uniform dose distribution compared to IMRT technique.

The prototype concept upon which this report was based required nondivergence central axis plane to abut each dose brick. Since all fields are abutted by nondivergence central axis plane, the junction plane should use jaw end surface instead of block or MLC shape surface. Although radiation field can be piled up by nondivergent central axis plane to focus the radiation dose on the lesion, normal tissue will obtain undesired radiation dose because the radiation beam passes through the normal tissue before or after the beam penetrates the lesion. However, normal tissues dose can be shared by multiple noncoplanar beams. Critical organs sparing was taken to the premier place and lesion being irradiated by radiation to the utmost was the key point in performing dose bricks concept treatment planning successfully. Treatment planning computer system must have the ability to prescribe dose individually to each field and the accumulated dose of all fields should be renormalized to one point for isodose curve distribution demonstration. The prescribed dose reference point of RSAO and LSAO is the point located inside the overlapping shape and at about the geometry center of “A.” As for RAO and LAO, the dose reference point is also located at about the geometry center of fields RAO and LAO. SIV dose reference point has to be separated into two parts inside the lower portion of the bell shape. The total accumulated dose was then normalized to the geometry center of CTV. All of these dose reference points should be fine-tuned to form optimal dose coverage to the target. Usually these five fields described in dose bricks technique can achieve the CTV uniform dose requirement, but the number of fields including gantry, collimator, and couch rotation angle should be adjusted case by case.

For small field such as SIV, dose delivery verification was carried for comparison with regular external beam and can use TLDs in Rando phantom for spot check only. Dose combination verification of all fields can only use TLDs in Rando phantom for point dose check too. The contribution of SIV provides a significant compensation dose to the lower portion of bell shape lesion thus escalating the normal brain tissue dose as well; however, the area of 50% dose area is acceptable in clinical concern.

As for the penumbra of abutting field in air in setup condition, the results can only show the dose overlap with or without separation in air and cannot conclude for dose overlap quantity in tissue. The scatter dose affected the result more significantly in phantom than in air.

The white dash line showed on films of Figures 8 and 9 were an indication of mechanical rotational isocenter accuracy check. If the film check on Figures 8 and 9. The white dash line did not perform a uniform straight line, then the rotation isocenter of couch, gantry, and collimator may be an error in digital and mechanical display or malfunction due to gear depletion. The mechanical isocenter coincidence items in gantry, collimator, and couch were strongly suggested to be controlled within 1 mm to ensure dose delivery accuracy.

**Figure 12:**
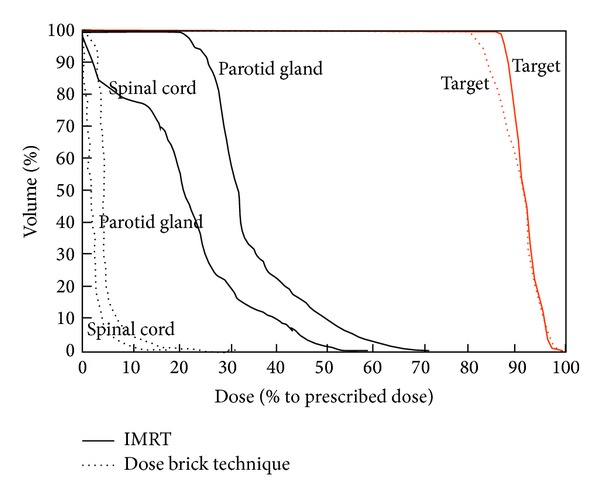
Comparison of DVH in DB technique and conventional IMRT.

Comparing the DVH of dose bricks to that of IMRT result, we find 50% volume of parotid glands receiving less than 10% of prescribed dose in dose bricks technique while the other result receives almost 35% in IMRT technique (Figure 12).

Measurements will be made of the dose distributions delivered using dose bricks patterns and compared by polymer gel with the MRI dose distributions. Further work is also needed on the verification of three-dimensional dose distribution delivered by this technique. This is a complex problem and will require rather extensive studies to provide conclusive evidence that whether the dose bricks concept in NPC treatment panning is functioning properly in all head and neck cases or only for individual patient. Comprehensive quality assurance of dose bricks technique in treatment planning is a crucial and complex issue and is beyond the scope of this preliminary investigation. Films exposure by different treatment setup in air was the only way to proof the rotational isocenter coincidence of the treatment facility. The importance of considerations of patient movement and positioning [39] and methods to assure that dose bricks concept in dose delivery are recognized as key issues.

## 5. Conclusion

NPC bell shape like lesion treatment planning method is based on dose bricks concept which can provide maximum target dose and minimize dose of critical organs, especially in parotid glands. According to the film verification results of experimental setup, field matching can be verified and adjusted before treatment. In dose bricks approach, dose delivery can be checked by spot only currently, the three-dimensional dose verification system is developed such as polymer gel, and the accuracy of dose delivery can be verified more precisely and easily.

Dose bricks concept in NPC treatment planning is an alternative approach not only in much lower critical organs dose especially in parotid glands but also when compared to the expensive facility in IMRT. Compared with IMRT, the expenditure of planning time and costing, “dose bricks” may after all be accepted as an optional implementation in nasopharyngeal carcinoma conformal treatment plan. This concept not only is suitable for head and neck lesion but also fits the need of other part lesions if organ sparing and noncoplanar technique can be executed.

## Figures and Tables

**Table 1:** The overall beam parameters in Dose Bricks concept treatment planning.

Parameter/field size	RSAO	LSAO	LAO	RAO	SIV
Prescribe dose (cGy)	90	90	45	45	25
Reference point	RSAO/LSAO^a^	RSAO/LSAO^b^	LAO^c^	RAO^d^	SIV^e^
Relative weighting	50%	50%	100%	100%	100%

Gantry angle	260	98	180	172	261
Couch angle	120	250	180	180	90
Collimator angle	180	172	153	199	175
Field size (*Y*_1_/*Y*_2_ × *X*_1_/*X*_2_)	6/2 × 2/4	6/2 × 5/2	6/1 × 2/5	9/1 × 5/2	4/4.5 × 3/1
Wedge/Orientation	60°/out^f^	60°/out	45°/left^g^	45°/right^h^	

a~e: the abbreviation of a~e see contains in the text.

f: wedge orientation out means “thick head” toward gantry side.

g: wedge orientation left means “thick head” toward patient right side.

h: wedge orientation right means “thick head” toward patient left side.
